# Supplementary material for: Innovative Therapeutic Delivery of Metastasis-Associated in Colon Cancer 1-Suppressing miRNA Using High Transmembrane 4 L6 Family Member 5-Targeting Exosomes in Colorectal Cancer Mouse Models
Source: Int J Mol Sci. 2024 Aug 26;25(17):9232. doi: 10.3390/ijms25179232 (PMC11394864; doi:10.3390/ijms25179232)

Supplementary Figure S1. Short Tandem Repeat (STR) identification data for cell lines used.

Markers	HT29	HCT116	SW480
D3S1358	15,17	12,19	15
vWA	17,19	17,22	16
FGA	20,22	18,23	24
Amelogenin	X	X,Y	X
TH01	6,9	8,9	8
TPOX	8,9	8	11
CSF1PO	11,12	7,10	13,14
D5S818	11,12	10,11	13
D13S317	11, 12	10,12	12
D7S820	10	11,12	8

Supplementary Figure S2. Western blot analysis demonstrating the impact of tEx[miR-143] on apoptotic markers in additional colorectal cancer cell lines. (A) Western Blot Analysis in HT29 cells under different treatment conditions: Ct, Ex, Ex[miR-940], tEx[miR-940], Ex[miR-143], and tEx[miR-143]. The bar graphs represent the relative density of MACC-1, Bcl-xL, and PUMA protein levels in HT29 cells under different treatment conditions. The tEx[miR-143] group showed the lowest levels of MACC-1 and BCL-xL and the highest levels of PUMA. (B) Western Blot Analysis in SW480 cells under different treatment conditions: Ct, Ex, Ex[miR-940], tEx[miR-940], Ex[miR-143], and tEx[miR-143]. The bar graphs represent the relative density of MACC-1, Bcl-xL, and PUMA protein levels in HT29 cells under different treatment conditions. The tEx[miR-143] group showed the lowest levels of MACC-1 and BCL-xL and the highest levels of PUMA. Values are presented as mean ± standard deviation of three independent experiments. * $P < 0.05$.

Figure S2

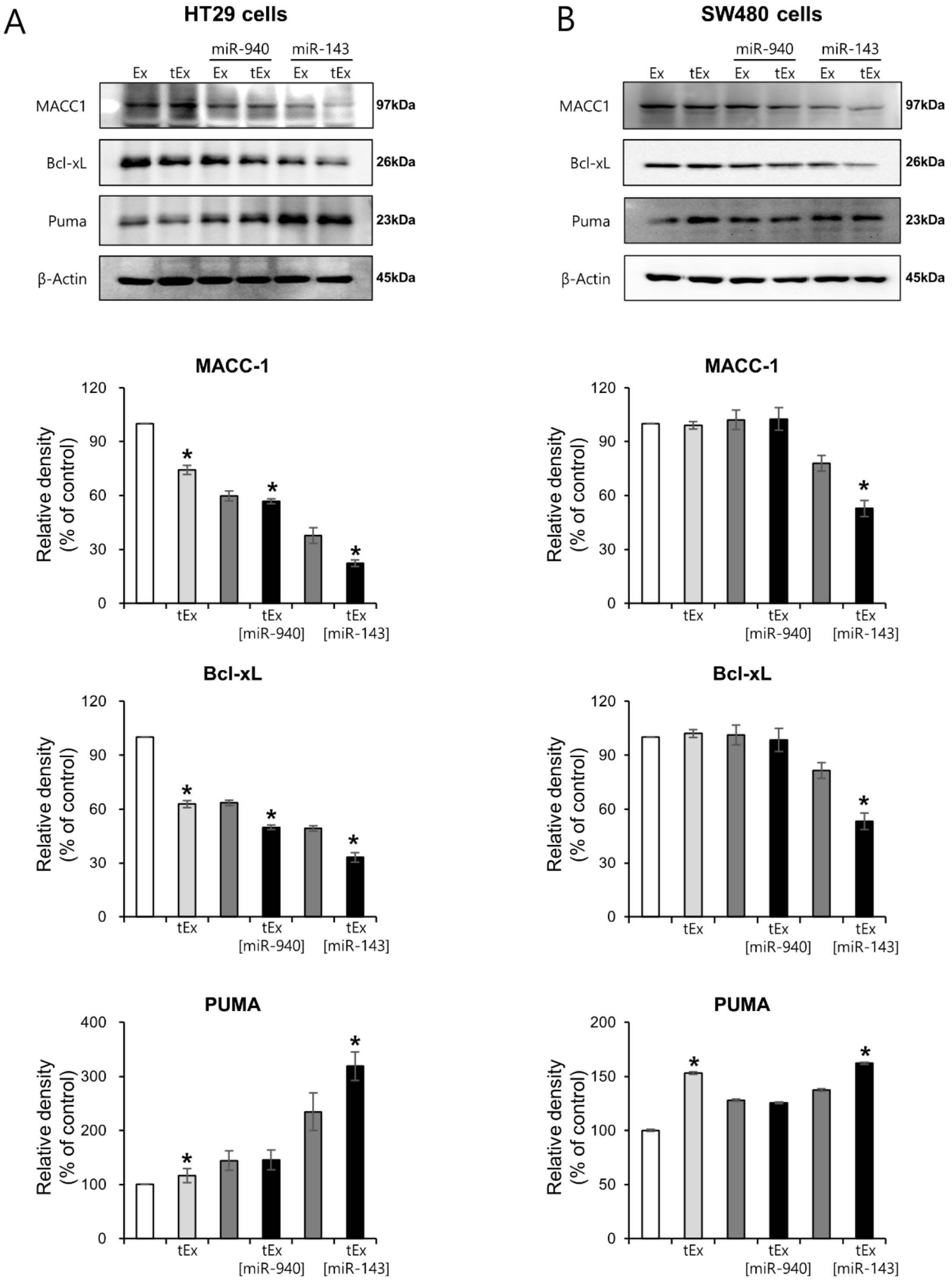
A
HT29 cells
miR-940
miR-143
Ex
tEx
MACC1
Bcl-xL
Puma
β-Actin
97kDa
26kDa
23kDa
45kDa
B
SW480 cells
MACC-1
Bcl-xL
PUMA
Relative density
(% of control)
tEx
tEx [miR-940]
tEx [miR-143]
*

Supplementary Figure S3. Cell viability in various CRC cell lines and doxorubicin-resistant CRC cell lines following Treatment with Ex[miR-143] and tEx[miR-143]. (A) MTT assay evaluating of cell viability in SW480, HT29, and HCT116 cell lines Following treatment with Ex[miR-143] and tEx[miR-143]. (A) An MTT assay showing the cell viability in SW480, HT29, and HCT116 cell lines following each treatment. The treatment with tEx[miR-143] resulted in a significant reduction in cell viability in SW480 and HCT116 cells compared to Ex[miR-143] ($P < 0.05$). In HT29 cells, tEx[miR-143] treatment also led to a reduction in cell viability compared to Ex[miR-143], although the difference was not statistically significant. Overall, tEx[miR-143] treatment decreased cell viability in all tested cell lines. (B) The effect of Ex[miR] and tEx[miR] treatments on the viability of doxorubicin-resistant CRC cells. HCT116-R and TH29-R cells. The tEx[miR] treatment significantly decreased the viability of both HCT116-R and TH29-R cells compared to the Ex[miR] treatment at both time points ($P < 0.05$). Values are presented as mean ± standard deviation of three independent experiments. * $P < 0.05$.

Figure S3

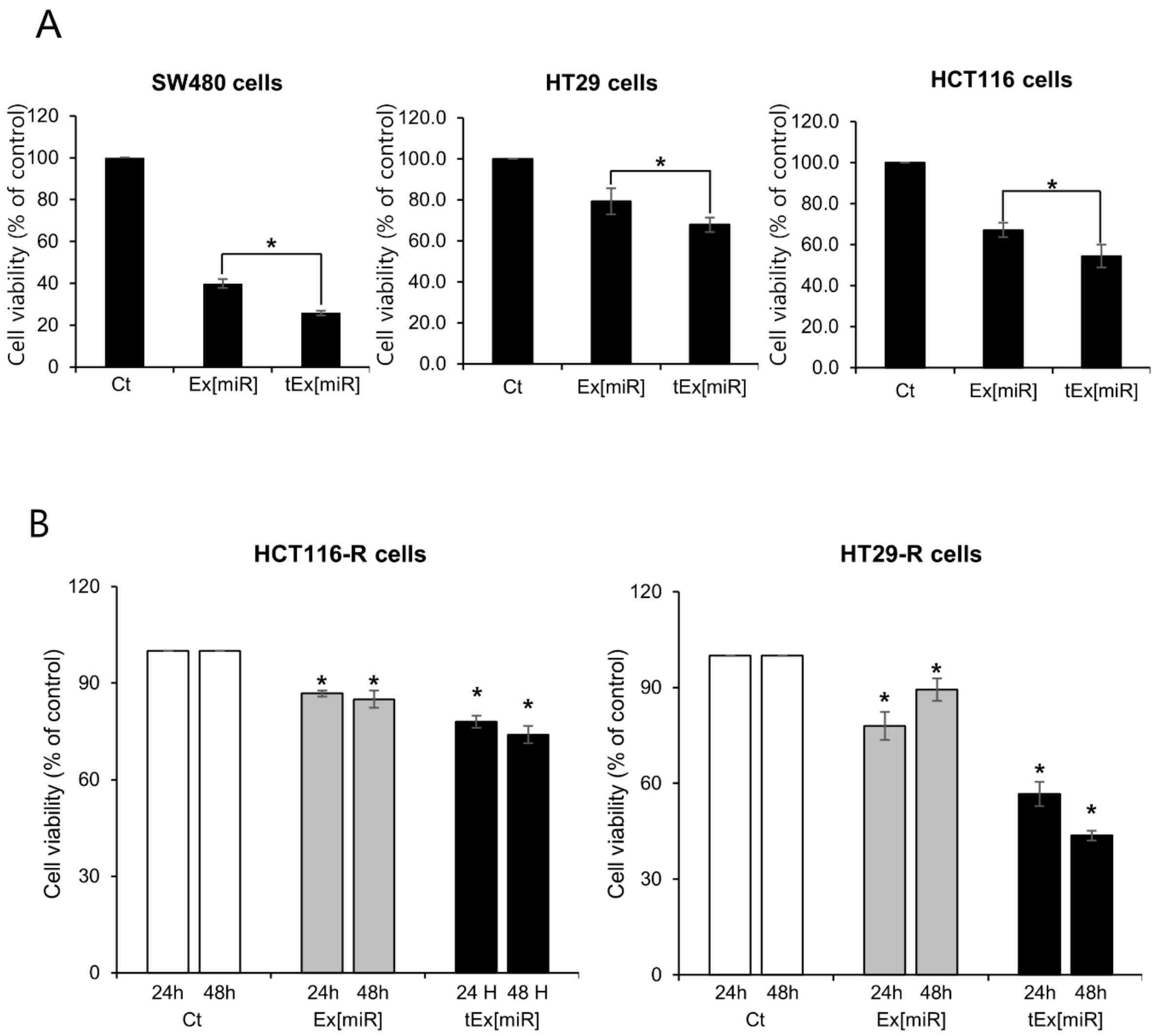
A
SW480 cells
HT29 cells
HCT116 cells
Cell viability (% of control)
Ct
Ex[miR]
tEx[miR]
B
HCT116-R cells
HT29-R cells
Cell viability (% of control)
24h 48h
24 H 48 H
Ct
Ex[miR]
tEx[miR]

Supplementary Figure S4. Wound healing assay demonstrating the Effect of tEx[miR] on cell migration and invasion in HT29 and SW480 colorectal cancer cell lines. (A) Representative images of wound healing assays in HT29 colorectal cancer cell lines treated with different conditions: Ct, Ex, Ex[miR], tEx, and tEx[miR]. Images were captured at 0 hours and 48 hours post-treatment. The tEx[miR] group exhibited the most significant inhibition of wound closure compared to the other groups, indicating reduced cell migration and invasion. Bar graphs showing the quantification of cell invasion as a percentage of the initial wound area in HT29 cells after 48 hours of treatment. (B) Representative images of wound healing assays in SW480 colorectal cancer cell lines treated with different conditions. The tEx[miR] group exhibited the most significant inhibition of wound closure compared to the other groups, indicating reduced cell migration and invasion. Values are presented as mean ± standard deviation of three independent experiments. * $P < 0.05$.

Figure S4

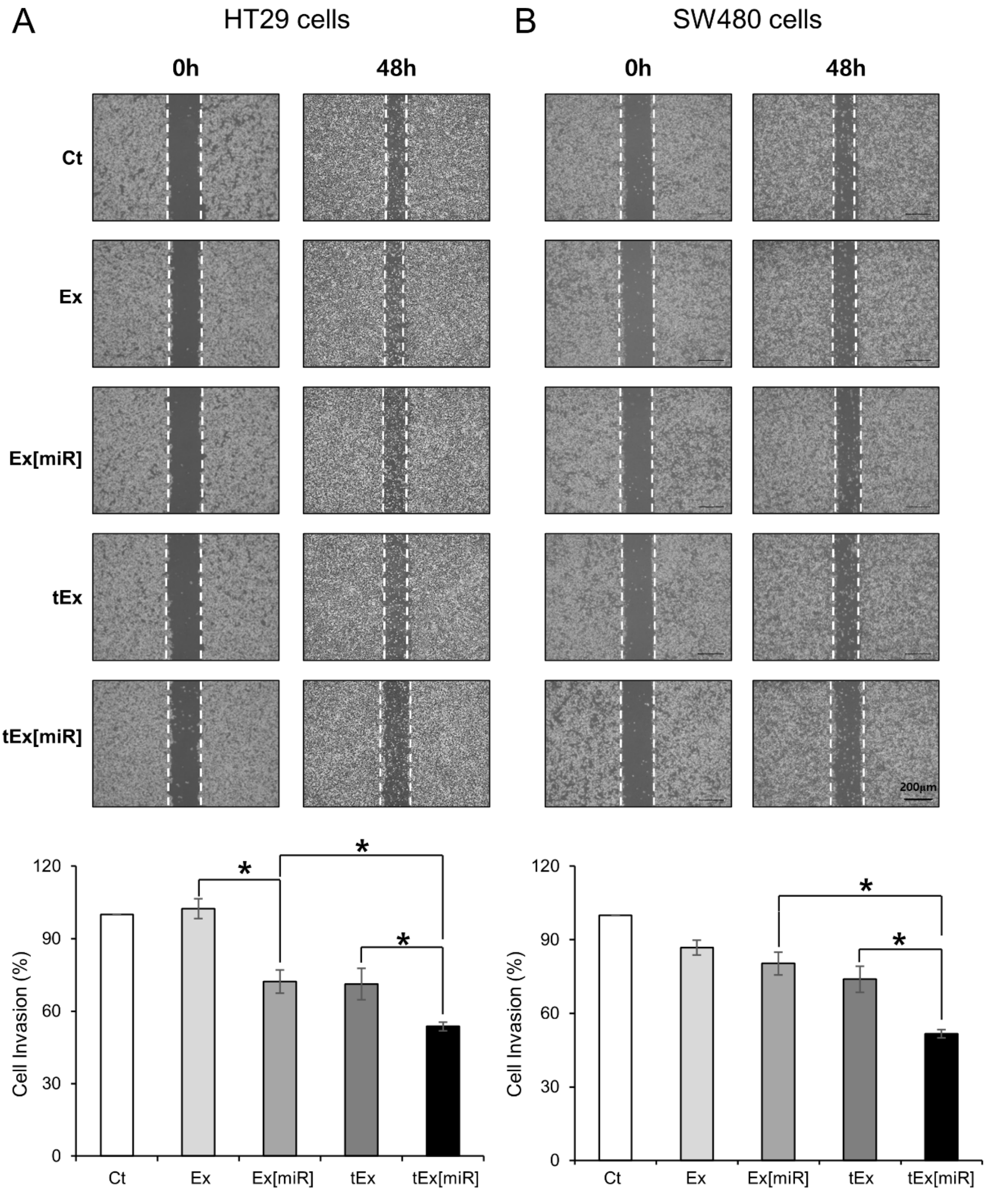
A
HT29 cells
B
SW480 cells
0h
48h
0h
48h
Ct
Ex
Ex[miR]
tEx
tEx[miR]
200μm
Cell Invasion (%)
120
90
60
30
0
*
Ct
Ex
Ex[miR]
tEx
tEx[miR]
Cell Invasion (%)
120
90
60
30
0
*
Ct
Ex
Ex[miR]
tEx
tEx[miR]

Supplementary Figure S5. Immunofluorescence analysis of EMT marker expression altered by various Ex formulations in HT29 and SW480 colorectal cancer cell lines. (A) Immunofluorescence analysis in HT29 cells. Immunofluorescence analysis determining the expression levels of E-cadherin (red), Snail (green), and Vimentin (red) in HT29 colorectal cancer cell lines treated with different conditions: Ct, Ex, Ex[miR], tEx, and tEx[miR]. Nuclei are stained blue with DAPI. The relative density of each marker is quantified in the bar graphs below the images. The tEx[miR] group exhibited significantly increased E-cadherin and decreased Snail and Vimentin levels in HT29 cells ($Ps < 0.05$), indicating a significant inhibition of EMT. (B) Immunofluorescence analysis in SW480 cells. The tEx[miR] group exhibited significantly increased E-cadherin and decreased Snail and Vimentin levels in SW480 cells ($Ps < 0.05$), indicating a significant inhibition of EMT. Values are presented as mean ± standard deviation of three independent experiments. *$P < 0.05$.

Figure S5

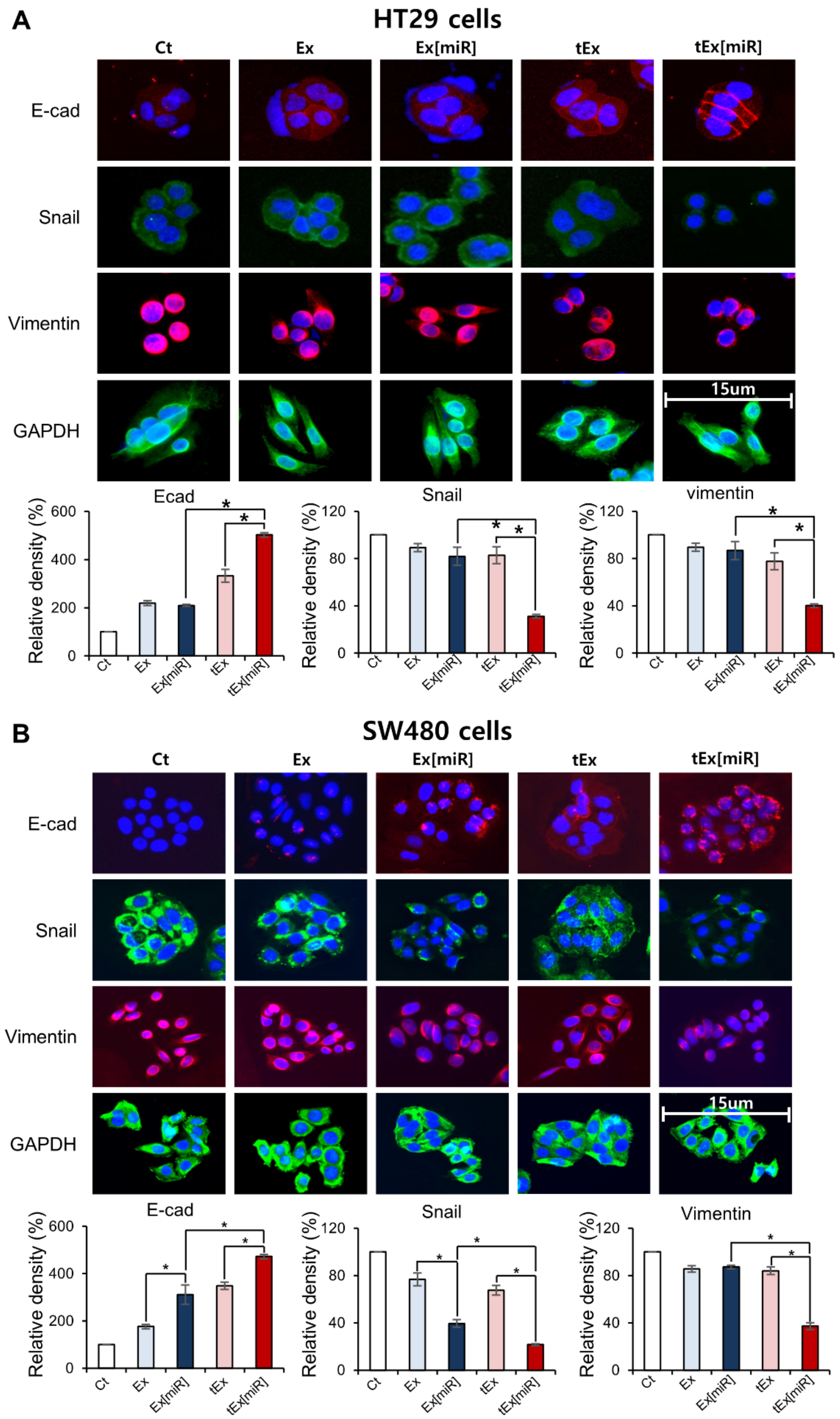
A
HT29 cells
Ct
Ex
Ex[miR]
tEx
tEx[miR]
E-cad
Snail
Vimentin
GAPDH
15um
Ecad
Snail
vimentin
Relative density (%)
B
SW480 cells
E-cad
Vimentin

Supplementary Figure S6. Impact of exosomal treatments on spheroid growth and cell viability. Spheroid cultures were used to simulate the tumor microenvironment and assess the impact of exosomal treatments on ECM factors and cell viability. The images show the progression of spheroid growth over a period of 5 days under different treatment conditions: Control (Ct), Exo[miR], and tExo[miR]. In the control group, spheroids were untreated. In the Ex[miR] group, spheroids were treated with exosomes loaded with miR-143. In the tExo[miR] group, spheroids were treated with targeted Ex loaded with miR-143. Cell viability was assessed using a LIVE/DEAD cytotoxicity assay (Invitrogen) following a 30-minute incubation at 37°C. Compared to the Ex[miR] treatment group, the tEx[miR] treatment group showed a significantly increased dead cell/live cell ratio ($P < 0.05$). This indicates that tEx[miR] treatment induces higher cell death in the tumor microenvironment compared to Ex[miR].

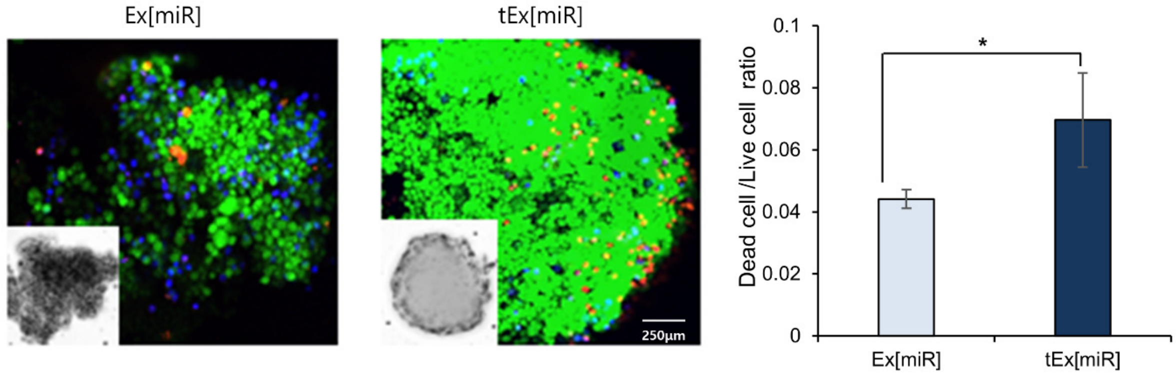

Supplement: Supplementary file 1 [file ijms-25-09232-s001.zip › ijms-3114635-supplementary.pdf]
